# Injury and fatality risks for child pedestrians and cyclists on public roads

**DOI:** 10.1186/s40621-024-00497-2

**Published:** 2024-04-11

**Authors:** David I. Swedler, Bina Ali, Rebecca Hoffman, Jennifer Leonardo, Eduardo Romano, Ted R. Miller

**Affiliations:** 1 UMass Chan Medical School, 55 N Lake Ave, Worcester, MA 01655 USA; 2 Pacific Institute for Research and Evaluation, 4061 Powder Mill Road, Suite 350, Beltsville, MD 20705 USA; 3 Education Development Center, 300 Fifth Avenue, Suite 2010, Waltham, MA 02451 USA; 4 Curtin University School of Public Health, Kent St, Bentley, WA 6102 Australia

**Keywords:** Motor vehicle crashes, Roadway factors, Circumstances, Vehicle type, Fatal injury, Nonfatal injury

## Abstract

**Background:**

Pedestrians and cyclists are often referred to as “vulnerable road users,” yet most research is focused on fatal crashes. We used fatal and nonfatal crash data to examine risk factors (i.e., relationship to an intersection, urbanicity, crash circumstances, and vehicle type) for police-reported pedestrian and cyclist injuries on public roads among children aged 0–9 and aged 10–19. We also compared risk factors among these two age groups with adults aged 20–29 and aged 30–39.

**Methods:**

Crash data were obtained for 2016–2020 from the National Highway Traffic Safety Administration’s Fatality Analysis Reporting System for fatal crash injuries and Crash Report Sampling System for nonfatal crash injuries. We collected data on victim demographics, roadway, and vehicle- and driver-related factors. Descriptive analyses were conducted between and within pedestrian and cyclist victims.

**Results:**

We analyzed 206,429 pedestrian injuries (36% in children aged 0–19) and 148,828 cyclist injuries (41% in children aged 0–19) from 2016 to 2020. Overall, child pedestrians had lower injury rates than adults, but children aged 10–19 had greater cycling crash rates than adults. Almost half of the pedestrian injuries in children aged 0–9 were “dart-out” injuries (43%). In the majority of the cyclist injuries, children in both age groups failed to yield to vehicles (aged 0–9 = 40% and aged 10–19 = 24%). For children and all ages included in the study, the fatality risk ratio was highest when pedestrians and cyclists were struck by larger vehicles, such as trucks and buses. Further exploration of roadway factors is presented across ages and transportation mode.

**Conclusion:**

Our findings on child, driver, vehicle, and roadway factors related to fatal and nonfatal pedestrian and cyclist injuries may help to tailor prevention efforts for younger and older children.

**Supplementary Information:**

The online version contains supplementary material available at 10.1186/s40621-024-00497-2.

## Background

Motor vehicle crashes (MVCs) on public roads account for over 40,000 deaths in the United States each year, and 22% of these deaths are a result of pedestrian and pedal-cyclist injuries (Centers for Disease Control and Prevention 2022). Though walking and cycling are often encouraged as simple and cost-effective ways of being active and benefiting the environment (e.g., reduced emissions of air pollutants, greenhouse gases, and noise) (World Health Organization 2022), they pose a traffic safety challenge. In recent years, roads in the United States have become more dangerous for pedestrians and cyclists, a group sometimes referred to as “vulnerable road users” (U.S. Department of Transportation 2022). Between 2011 and 2020, pedestrian fatalities increased over 30% and cyclist fatalities increased by about 27%, for a total of 6,516 and 938 deaths for each group, respectively (National Center for Statistics and Analysis 2020a, b). Fatal and nonfatal pedestrian crashes accounted for approximately $17.6 billion in economic costs based on 2019 dollars, while cyclist crashes accounted for approximately $5.6 billion in economic costs (Blincoe et al. 2019). These are cost increases of 27% and 16%, respectively, over pedestrian and cyclist crashes in 2010 (Blincoe et al. 2010) (inflated to 2019 dollars).

Many studies have explored factors associated with pedestrian and cyclist safety, including drivers’ behaviors (e.g., distraction, drinking and driving), driver and pedestrian education, and built environment (Asgarzadeh et al. 2017; Glass et al. 2014; Saadati et al. 2022; Stoker et al. 2015; Useche et al. 2018), and conducted reviews of existing interventions to promote road safety (Fisa et al. 2022; Kwan and Mapstone 2006). While there is extensive research on risk factors for MVCs in general, differences in risk factors between age groups are understudied. Human developmental stages and experiences with transportation contribute to variation in risk factors for different age groups. For example, very young children are not developed enough to operate a pedal-cycle, so their risk for crash injury would occur as a passenger in situations beyond their control (Oxley et al. 2016). In this case, the risks for injury would be impacted by cognitive and perceptual development and how that impacts their mobility in the road environment (Schwebel et al. 2012). As children age, they can be cyclists and mobile enough to be pedestrians, while gaining independence, making their own decisions, and engaging in more varied behaviors as pedestrians and cyclists (Abdel-Aty et al. 2007). Vulnerable adult road users, on the other hand, are more likely to be at risk due to other behaviors, such as walking or riding while distracted in other ways or under the influence of drugs or alcohol (Asbridge et al. 2014; Plurad et al. 2006). Thus, there is a need to examine the differential risk factors for MVCs among different age groups. Additional knowledge could be used to tailor prevention programs targeting MVCs among vulnerable child road users.

Another gap in the literature is that historically, most U.S. research on non-occupant crashes has focused on fatal crashes. Studies often provide detailed breakdowns about crash fatalities and give a single table about survivors (e.g., fatality rate by age group), or draw nonfatal counts from medical data detailing injury location and severity rather than National Highway Traffic Safety Administration (NHTSA) crash datasets detailing crash circumstances (McAdams et al. 2018; Mehan et al. 2009; Miller et al. 2004; Wheeler-Martin et al. 2017). Using NHTSA databases that provide fatal (Fatality Analysis Reporting System, FARS) and nonfatal (Crash Report Sampling System, CRSS) crashes on public roads, this study described MVC injury hazards and rates for young pedestrians and cyclists in the United States from 2016 to 2020. The data excluded injuries in driveways, parking lots, and off-road areas like beaches and farms. We compared children under age 10 to those aged 10 through 19. We also compared these two age groups with adults aged 20–29 and aged 30–39 to identify risks that are unique to vulnerable child road users.

## Methods

### Data sources

We identified fatal crash injuries in 2016–2020 from FARS, giving us a complete picture of all pedestrian and cyclist fatalities (National Center for Statistics and Analysis 2022c). For nonfatal injuries, we used CRSS to identify only nonfatal pedestrian and cyclist injuries. CRSS is a weighted sample of all police-reported crashes in the United States, capturing MVCs outside the scope of the FARS census of fatal crashes (NHTSA 2022).

In FARS and CRSS, we identified crashes involving at least one injured or killed pedestrian or cyclist by using the Person Type variable from the Person file. From the Person file, we selected all injured pedestrians and cyclists under age 40 and assigned them to the 0–9, 10–19, 20–29, and 30–39 age categories. We then used the Person Number and Crash Number to identify pedestrian- and cyclist-specific crash elements. Additional file 1: Table S1 in the additional file describes how crash types from the pedestrian and cyclist variables were recategorized for categorical crash circumstance analysis (see Additional file 1). Crash Number was used to select further data elements from the Vehicle and Accident files in each year for crashes involving these vulnerable road users. Additional file 1: Table S2 in the additional file displays how we recategorized the FARS and CRSS vehicle type variables into analytic categories for this investigation (see Additional file 1). We downloaded the annual Population Estimates Program county-level population data for 2016–2020 from the U.S. Census Bureau (U.S. Census Bureau 2021) to calculate crash rates per 100,000 population. Urban–Rural Continuum Codes were obtained from the U.S. Department of Agriculture (U.S. Department of Agriculture 2020) to analyze urban and rural populations.

### Analysis

Our main descriptive analysis was to calculate injury rates for younger and older children by year, relationship to an intersection, urbanicity, crash circumstances, and vehicle type. We then compared the injury frequencies, and rates per 100,000 population to the two adult age blocks, to assess if and how the injury situations varied by age, and if certain risk factors were unique to younger vulnerable road users. We compared injury frequencies for each age group by vehicle type and crash circumstances using the *χ*^2^ statistic. Because it already is established that male vulnerable road users are injured and killed at much greater rates than female vulnerable road users (Coleman and Mizenko 2018), we did not include an analysis of age and gender effects.

Further, we compared crash fatality risk across age groups. Risk ratios (RRs) were calculated for decade of age (with aged 0–9 as reference group for analyses for children and with aged 20–29 as reference group for analysis for all ages), vehicle type (with Cars as reference group), crash circumstances (with Crossing as reference group for pedestrians and Intersection- Motorist Failed to Yield as reference for cyclists), and street location (with Intersection as reference group). Because the FARS crashes are from a complete census and CRSS injuries are estimates, bootstrapped 95% confidence intervals (CIs) were generated for the RRs. Finally, we tested for interactions of age and vehicle type, age and crash circumstances, and age and street location using RRs. This study did not meet the definition of human subjects research and therefore was not subject to institutional review board (IRB) review.

## Results

### Descriptive results

**Fig. 1:**
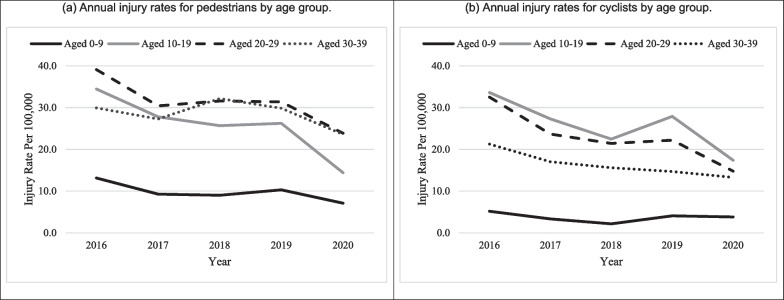
Annual injury rates for **a** pedestrians and **b** cyclists by age group

Between 2016 and 2020, the study observed 206,429 pedestrian injuries (36% in children aged 0–19) and 148,828 cyclist injuries (41% in children aged 0–19). Among children aged less than 20, boys (51%) and girls (49%) had similar pedestrian injuries, but cyclist injuries were greater for boys (81%) than girls (19%), suggesting riskier cycling behaviors among boys. Table 1 displays the frequencies involving injured pedestrians and cyclists by victim, roadway, crash scenario, and vehicle characteristics. The percentage of fatal injuries was greater for pedestrians involved in crashes compared to cyclists (Children aged 0–19: 3% vs. 1%, respectively; and All ages: 6% vs. 1%, respectively). Across all age groups, fatality risks were greater when pedestrians and cyclists were injured by trucks and buses and injured in rural areas and at non-intersections. While crash injuries decreased in 2020, fatality risk was highest in 2020 for both pedestrians and cyclists. Figure 1 shows a decrease in injury rates for pedestrians and cyclists from 2016 through 2020 for children and adults. Child pedestrians had lower injury rates per 100,000 population than the two adult age groups (shown in 1a), and children aged 10–19 had greater cycling crash rates than adults each year (shown in 1b).

**Table 1:** Description of pedestrian and cyclist crashes in the United States, 2016–2020

	Pedestrians aged 0–19		Cyclists aged 0–19		Pedestrians aged 0–39		Cyclists aged 0–39	
	Frequency	Percent fatal (%)	Frequency	Percent fatal (%)	Frequency	Percent fatal (%)	Frequency	Percent fatal (%)
Total	73,369	2.9	61,278	0.7	206,429	5.6	148,828	1.0
*Sex*								
Male	34,778	3.8	49,192	0.8	114,679	6.9	117,413	1.2
Female	33,279	2.4	11,913	0.3	91,104	3.8	31,157	0.9
*Age*								
0–9	19,554	3.2	7429	1.1	19,554	3.2	7429	1.1
10–19	53,815	2.8	53,849	0.7	53,815	2.8	53,849	0.7
20–29	–	–	–	–	70,744	6.3	51,857	0.9
30–39	–	–	–	–	62,316	7.9	35,693	1.5
*Year*								
2016	19,699	2.6	16,133	0.6	50,179	4.5	39,926	0.7
2017	15,380	3.0	12,777	0.8	40,969	5.4	30,872	0.9
2018	14,376	2.9	10,294	0.9	42,758	5.5	26,834	1.1
2019	15,091	2.4	13,314	0.6	42,467	5.3	29,847	1.0
2020	8823	4.2	8760	1.1	30,056	8.1	21,319	1.4
*Vehicle type*								
Car	40,121	2.4	32,314	0.6	108,947	5.0	79,626	0.8
Van/utility vehicles/light truck	26,385	3.7	23,862	0.9	72,273	6.4	46,819	1.2
Truck/bus	1691	8.5	1115	5.0	5916	16.5	3741	5.1
Other	5365	3.0	8586	0.3	16,981	7.9	14,556	0.9
*Urbanicity*								
Urban	60,354	2.6	51,875	0.6	174,140	5.1	130,752	0.8
Rural	12,985	3.9	9396	1.5	32,218	8.1	18,033	1.9
*Street location*								
Intersection	36,984	1.3	41,654	0.5	104,763	2.0	41,654	0.5
Non-intersection	36,384	4.5	19,623	1.4	101,664	9.3	19,623	1.4
*Circumstances–pedestrians*								
Crossing	34,416	1.4	–	–	101,974	2.9	–	–
Dart out	18,411	1.5	–	–	28,735	2.7	–	–
Walk/run along roadway	4295	6.0	–	–	16,415	10.1	–	–
Unusual circumstances	3683	5.0	–	–	16,791	6.5	–	–
Other	12,249	4.5	–	–	40,595	7.6	–	–
*Circumstances–cyclists*						–	–	
Intersection-motorist	–	–	6234	0.1	–	–		
Intersection-cyclist	–	–	11,200	0.1	–	–	20,743	0.9
Crossing paths	–	–	11,290	0.3	–	–	23,961	0.3
Midblock-cyclist	–	–	4645	0.8	–	–	7047	1.1
Motorist turn	–	–	6749	0.3	–	–	23,323	0.3
Other	–	–	21,076	1.0	–	–	58,300	1.2

Table 2 examines incidence by age group for vehicle type, urbanicity, intersection involvement, and crash circumstances. All *χ*^2^ in Table 2 identify significant differences at the level of *p* < 0.001. Cars were the most common vehicle involved in pedestrian and cyclist crashes, accounting for approximately half of the injured victims in each age group. The biggest difference by vehicle type was that child cyclists aged 0–9 were more likely to be injured by vans, utility vehicles, and light trucks (44%) than child cyclists aged 10–19 (35%) and both groups of adult cyclists aged 20–29 (23%) and aged 30–39 (36%). For pedestrian crash circumstances, we observed 43% of children 0–9 were injured in dart-out crashes, compared to 19% of children 10–19, and 8% and 7% of adults 20–29 and 30–39, respectively. For cyclist crash circumstances, we again found a different pattern for the youngest children: over 26% of injuries to cyclists aged 0–9 were due to midblock-cyclist failed to yield crashes compared to 5% of children 10–19, and 3% in both adult age groups. Adult cyclists were more likely to be injured in motorist turn (left or right) crashes (approximately 19% for both age groups) compared to children aged 0–9 (6%) and aged 10–19 (12%).

**Table 2:** Distribution of vehicle type and crash circumstances by age group for pedestrians and cyclists

	Pedestrians					Cyclists				
	Aged 0–9 (%)	Aged 10–19 (%)	Aged 20–29 (%)	Aged 30–39 (%)	Chi-square test	Aged 0–9 (%)	Aged 10–19 (%)	Aged 20–29 (%)	Aged 30–39 (%)	Chi-square test
*Vehicle*										
Car	56.3	53.9	53.2	52.1	*χ*^2^ = 735 (df = 9), *p* < 0.001	48.9	49.1	64.4	54.7	*χ*^2^ = 5191 (df = 9), *p* < 0.001
Van/utility vehicles/light truck	4.2	8.4	8.3	9.6		44.4	35.2	23.2	36.3	
Truck/bus	2.3	2.3	3.3	3.2		2.3	1.6	4.0	2.5	
Other	37.2	35.4	35.2	35.1		4.4	14.1	8.5	6.5	
*Urbanicity*										
Urban	82.4	82.3	85.5	85.6	*χ*^2^ = 13,532 (df = 3), *p* < 0.001	77.3	85.7	90.2	90.0	*χ*^2^ = 33,558 (df = 3), *p* < 0.001
Rural	17.6	17.7	14.5	14.4		22.7	14.3	9.8	10.0	
*Street location*										
Intersection	38.4	54.8	52.5	49.2	*χ*^2^ = 14,885 (df = 3), *p* < 0.001	46.9	70.9	66.7	66.6	*χ*^2^ = 34,167 (df = 3), *p* < 0.001
Non-intersection	61.6	45.2	47.5	50.8		53.1	29.1	33.3	33.4	
*Circumstances*										
Crossing	31.7	52.7	51.7	51.0	*χ*^2^ = 20,418 (df = 12), *p* < 0.001	–	–	–	–	–
Dart out	42.5	18.9	8.4	7.2		–	–	–	–	
Walk/run along roadway	2.7	7.0	9.2	9.2		–	–	–	–	
Unusual circumstances	3.5	5.6	10.2	9.7		–	–	–	–	
Other	19.6	15.7	20.4	22.9		–	–	–	–	
*Circumstances–cyclists*										
Intersection-motorist failed to yield	–	–	–	–	–	3.3	11.1	9.2	11.6	*χ*^2^ = 31,995 (df = 15), *p* < 0.001
Intersection-cyclist failed to yield	–	–	–	–		13.9	18.9	11.0	10.9	
Crossing paths	–	–	–	–		21.6	18.0	15.7	12.7	
Midblock-cyclist failed to yield	–	–	–	–		26.2	5.0	2.6	2.9	
Motorist turn	–	–	–	–		6.0	11.7	19.3	18.6	
Other						29.0	35.2	42.2	43.3	

### Risk factors for pedestrian and cyclist injuries across age groups

Table 3 displays fatality risk ratios for vehicle type, urbanicity, street location, and crash circumstances for pedestrians and cyclists aged 0–19 and all pedestrians and cyclists aged 0–39. For injured child pedestrians and cyclists, cars had lower fatality risk than vans/utility vehicle/light trucks and trucks and buses, with cyclist injuries from trucks and buses having the greatest fatality risk (RR = 8.03). This risk pattern was similar for adult pedestrians and cyclists. Child cyclists struck by other vehicles, like motorcycles and mopeds, had the lowest risk of death (RR = 0.52). For all pedestrians and cyclists, fatality risk ratios were greater for crashes in rural areas versus urban areas, and those that occurred not at intersections versus those at intersections. Child cyclists aged 0–9 had greater fatality risk in crashes (RR = 1.33) than adults aged 20–29. Child pedestrians in both age groups (Children aged 0–9 RR = 0.51; Children aged 10–19 = 0.44) and cyclists aged 10–19 (RR = 0.86) had lower fatality risk in crashes compared to adults aged 20–29.

**Table 3:** Fatality risk ratios for pedestrians and cyclists crash injury by vehicle type and crash circumstances

	Pedestrians aged 0–19		Cyclists aged 0–19		Pedestrians aged 0–39		Cyclists aged 0–39	
	RR	95% CI	RR	95% CI	RR	95% CI	RR	95% CI
*Age*	–	–	–	–				
20–29	–	–	–	–	1.0		1.0	
0–9	–	–	–	–	0.51	(0.46, 0.55)	1.33	(1.04, 1.65)
10–19	–	–	–	–	0.44	(0.44, 0.47)	0.86	(0.75, 0.98)
30–39	–	–	–	–	1.25	(1.20, 1.29)	1.82	(1.61, 2.06)
*Vehicle*								
Cars	1.0		1.0		1.0		1.0	
Vans/utility vehicles/light trucks	1.57	(1.43, 1.71)	1.39	(1.15, 1.69)	1.27	(1.23, 1.32)	1.66	(1.48, 1.85)
Trucks/buses	3.60	(3.02, 4.24)	8.03	(5.91, 10.62)	3.27	(3.06, 3.48)	6.42	(5.44, 7.47)
Other	1.28	(1.08, 1.50)	0.52	(0.33, 0.75)	1.56	(1.47, 1.65)	1.13	(0.93, 1.35)
*Urbanicity*								
Urban	1.0		1.0		1.0		1.0	
Rural	1.49	(1.35, 1.64)	2.39	(1.96, 2.89)	1.59	(1.52, 1.66)	2.22	(1.96, 2.51)
Street location								
Intersection	1.0		1.0		1.0		1.0	
Non-intersection	3.45	(3.13, 3.83)	2.99	(2.50, 3.61)	4.62	(4.41, 4.83)	3.45	(3.10, 3.83)
*Circumstances–pedestrians*								
Crossing	1.0		–	–	1.0		–	–
Dart out	1.08	(0.94, 1.25)	–	–	0.93	(0.85, 1.00)	–	–
Walk along roadway	4.19	(3.60, 4.85)	–	–	3.45	(3.25, 3.65)	–	–
Unusual circumstances	3.51	(2.97, 4.12)	–	–	2.23	(2.09, 2.39)	–	–
Other	3.17	(2.80, 3.57)	–	–	2.73	(2.59, 2.86)	–	–
*Circumstances–cyclists*								
Intersection-motorist	–	–	1.0		–	–	1.0	
Intersection-cyclist	–	–	9.02	(4.45, 45.64)	–	–	6.61	(4.46, 11.32)
Crossing paths	–	–	3.87	(1.78, 19.88)	–	–	0.50	(0.27, 0.95)
Midblock-cyclist	–	–	11.01	(5.18, 53.68)	–	–	8.29	(5.26, 14.59)
Motorist turn	–	–	3.51	(1.47, 17.55)	–	–	2.01	(1.27, 3.48)
Other	–	–	12.36	(6.21, 60.93)	–	–	8.96	(6.13, 15.23)

### Interaction of age with risk factors

Table 4 examines the interaction of age with vehicle type, street location, and crash circumstances on fatality risk. Due to small cell counts, a similar analysis of urbanicity was not pursued. For both pedestrians and cyclists, the reference group for age by vehicle type was adults aged 20–29 struck by cars (RR = 1). For age by crash circumstances, the reference group for pedestrians was adults aged 20–29 in crossing crashes, and for cyclists was adults aged 20–29 in intersection-motorist failed to yield crashes. The reference group for age by street location was adults aged 20–29 at intersections. In the age by vehicle interaction, children struck by buses and trucks had greater increased pedestrian (RR = 1.78 for children aged 0–9 and RR = 1.36 for children aged 10–19) and cyclist (RR = 11.39 for children aged 0–9 and RR = 6.28 for children aged 10–19) fatality risk compared to the adult reference group. For crash circumstances and age, the greatest increased fatality risk for child pedestrians was walking/running along roadway (RR = 2.03) and unusual circumstances (RR = 1.72) versus the adult reference group; however, this increase was only significant at *α* = 0.05 for children aged 10–19. For age by cyclist crash circumstances, compared to the adult reference group, the greatest increased fatality risk for children aged 10–19 was due to cyclist midblock-failure to yield (RR = 7.09) and other circumstances (RR = 5.41); for child cyclists aged 0–9, it was due to other circumstances (RR = 10.58).

**Table 4:** Fatality risk ratios from interactions of age and vehicle type and age and crash circumstances

	Pedestrians				Cyclists			
	Aged 0–9	Aged 10–19	Aged 20–29	Aged 30–39	Aged 0–9	Aged 10–19	Aged 20–29	Aged 30–39
*Vehicle*								
Cars	0.36	0.43	1.0	1.32	0.70	*0.90*	1.0	1.64
Vans/utility vehicles/light trucks	0.77	0.60	1.48	1.62	2.05	*1.10*	2.23	2.16
Trucks/buses	1.78	1.36	3.09	3.80	11.39	6.28	5.51	10.89
Other	0.70	0.49	1.51	1.89	*1.73*	0.41	2.00	3.05
*Street location*								
Intersection	0.85	0.62	1.0	1.47	2.15	0.85	1.0	1.43
Non-intersection	2.10	2.40	5.66	6.46	2.41	2.99	3.22	6.01
*Circumstances–Pedestrians*								
Crossing	0.69	0.42	1.0	1.48	–	–	–	–
Dart out	0.51	0.52	1.34	1.91	–	–	–	–
Walk along	*1.58*	2.03	3.39	4.32	–	–	–	–
Unusual circumstances	*1.38*	1.72	2.31	2.30	–	–	–	–
Other	1.99	0.69	2.84	3.09	–	–	–	–
*Circumstances–cyclists*								
Intersection-motorist	–	–	–	–	*4.91*	*0.30*	1.0	*1.16*
Intersection-cyclist	–	–	–	–	*3.47*	*0.36*	8.50	8.50
Crossing paths	–	–	–	–	*2.62*	*1.73*	3.17	3.17
Midblock-cyclist	–	–	–	–	*2.77*	7.09	13.81	13.81
Motorist turn	–	–	–	–	*5.39*	*1.42*	2.18	2.18
Other	–	–	–	–	10.58	5.41	6.39	10.96

RRs in italics are not significantly different from 1.0 at the *α* = 0.05 level

## Discussion

This study used fatal and nonfatal pedestrian and cyclist crash data from 2016 through 2020 to examine risk factors for younger children (aged 0–9) as compared with older children (aged 10–19), and to compare these two age groups with adults aged 20–29 and aged 30–39. Overall, we found that the fatality risk was greater for pedestrians involved in crashes compared to cyclists across all age groups, a finding consistent with previous reports (National Center for Statistics and Analysis 2020a, b). Consistent with research on pedestrian and cyclist injury risks across age groups based on health systems data, our analysis showed that children aged 10–19 had greater police-reported cyclist injury rates than other age groups in each year of our data, but both groups of child pedestrians had lower injury rates than adults aged 20–39. The present results align with prior reports from NHTSA that identify older adults as having greater pedestrian injury and fatality rates than children (National Center for Statistics and Analysis 2020a), and older children as having slightly greater cyclist injury and fatality rates than adults (National Center for Statistics and Analysis 2020b). While crash injuries decreased in 2020, fatality risk was highest in 2020 for both pedestrians and cyclists, which may be attributed to the disruption in healthcare services during the COVID-19 pandemic.

We found that dart-out crashes continue to be a unique hazard for the youngest pedestrians, consistent with previous research (Stevenson et al. 2015). When compared to risks of adults aged 20–29 when crossing, child pedestrians aged 0–19 darting out had lower risk of crash fatality, but the risk was greater for child pedestrians aged 10–19 walking/running along the roadway. A young child may dart out or run into the street to retrieve a toy or a ball or to cross the street with a lack of attention to the oncoming traffic. An older child may be cognitively capable of making traffic safety decisions, though they may misjudge speed, distance, and acceleration, and may engage in risky behaviors, such as walking while distracted or using their smartphones (Deluka-Tibljaš et al. 2022). We also found that the fatality risk was increased for child cyclists aged 0–19 due to cyclist midblock-failure to yield. A report on all-age pedestrian and cyclist fatalities identifies failure to yield right-of-way as the most common pedestrian and cyclist behavior associated with fatalities (Coleman and Mizenko 2018). Other common risk factors include being in roadway improperly (e.g., standing, lying, working) and invisibility for pedestrian fatalities, and invisibility and failure to follow traffic signs or signals for cyclist fatalities (Coleman and Mizenko 2018).

As vehicle mass went up, the likelihood of fatality increased, which we would expect to find. Heavier vehicles have been shown to have increased risk of mortality for pedestrians and cyclists (Tyndall 2021), though the effects of vehicle size are not consistent within and across age groups (Paulozzi 2005). It is not just the mass and unforgiving contact surfaces of these vehicles that creates the hazard for young children. As the U.S. vehicle fleet sees an increase in larger and taller light trucks and SUVs, front blind spots are making it increasingly difficult for drivers to see small children (Kaplan et al. 2022). This trend in increasing vehicle size has the potential to create further increased risks for vulnerable child road users in the future. However, a recent study on state-level pedestrian and cyclist fatalities demonstrated an inverse relationship between the number of vulnerable road users and pedestrian and bicyclist fatality, suggesting that there may be a ‘safety in numbers effect’ (Hafeez and Mehta 2021). This may be due to protective environmental factors, such as better infrastructure for walking and cycling, as well as increased awareness of vulnerable road users by motor vehicle drivers.

An important risk factor for vulnerable road users is higher speed, which increases the likelihood of being struck by a car and sustaining severe injuries (Tefft 2013). One report shows that most pedestrian deaths (60%) occur on high-capacity urban roads with posted speed limits of 45–55 miles per hour (Governors Highway Safety Association 2022). The driver of a fast-moving car has less time to respond to the pedestrian, and the distracted child pedestrian has less time to respond to the fast-coming car, thus increasing the risk of a severe injury or a fatal crash. Among children younger than age 15, speeding as a contributing factor of the pedestrian fatalities has doubled from 6 to 12% between 2018 and 2020 (Governors Highway Safety Association 2022), which warrants more attention. Also, the environmental conditions to ensure safety for children may be different than for adults given their limited developmental capacity to perceive road and traffic threats and their small size, which makes them less visible to drivers. In an immersive pedestrian simulation, O’Neal et al. (2018) studied road-crossing decisions of children at various ages relative to adults. They found that when confronted by a busy road, 8% of children aged 6, 6% of those aged 8, and 5% of those aged 10 made crossing decisions that resulted in their being “hit.” Adults were never hit, while the 2% hit rate at aged 12 was not significantly different from the adult rate. Their study suggests that the long-standing rule that children under age 8 should not be allowed to walk alone [e.g., Nidirect (n.d)] may be too liberal.

Employing all Three Es of injury prevention (Christoffel and Gallagher 1999), Education, Enforcement, and Engineering, is necessary to decrease child pedestrian and cyclist injuries. Education may include teaching parents, guardians, teachers, and providers on pedestrian and cycling about the United Kingdom’s green cross code or equivalent (Training 2023) and the difficulty children have in grasping its principles, as well as about roadway safety in general (e.g., using sidewalks, wearing a bicycle helmet, being visible and alert, obeying traffic rules, among other recommended behaviors) (National Center for Statistics and Analysis 2020a, b; National Highway Traffic Safety Administration 2008). Education and enforcement may also target driver behaviors, such as slowing down and yielding to pedestrians/cyclists, following speed limits, never driving under the influence of alcohol and/or drugs, and other recommended behaviors to ensure safety (National Center for Statistics and Analysis 2020a, b; Stevenson et al. 2015). Parental and adult supervision encourages children to walk slowly and engage in less risky behavior (Deluka-Tibljaš et al. 2022). Further, engineering can support changes to the built environment, including measures such as building sidewalks and well-lit streets, reducing traffic speed and volume, and adding speed bumps and roundabouts to reduce injury (Cloutier et al. 2021), as well as features of the motor vehicle (e.g., backup cameras, blind-spot detection, lane departure technology, and advanced sensor technology that can prevent collision).

### Limitations

Our analysis is limited to police-reported data available through NHTSA. In our comparison of the nonfatal data included in this study with the Healthcare Cost and Utilization Project (HCUP) hospitalization and emergency department visit data (data not presented as it is outside the scope of this study, but available upon request), we found that nonfatal pedestrian injuries are underreported by approximately 16% and cyclist injuries are underreported by approximately 37% in NHTSA. Data linkage efforts to integrate police reports with hospitalization data, as well as Medical Insurance Claims databases, Emergency Medical Systems data, and Vital Statistics, are recommended to comprehensively understand road safety (Cherry et al. 2018). Further, NHTSA only captures police-reported crashes on public roadways. While the majority of pedestrian injuries involve on-roadway collision with vehicle (about 67%), most cyclist crashes occur off-roadway (about 71%), according to HCUP nonfatal data (data not presented as it is outside the scope of this study, but available upon request). Future research may study the differential risks of pedestrian and cyclist injuries on roadway versus off roadway. In the study, missingness was not a major concern; however, we did not attempt to impute missing data in any of the tables in this manuscript given the descriptive nature of the study.

FARS and CRSS are long-running surveillance systems, and our analysis included variables that have been collected over time. Most variables in these datasets are consistent over time and across datasets, but not all. A change in measuring vehicle mass in 2020 limits the analysis of the effects of vehicle mass on injury severity. The NHTSA Crash Investigation Sampling System database has more precise data on crash vehicle mass; however, it was not designed to capture data on injured vulnerable road users (Radja et al. 2021). Our analysis did not further probe the severity of injuries within CRSS. Future research may consider these injury risk factors as they relate to injury severity.

We acknowledge that road use and mobility patterns change across the age spectrum, but we were unable to quantify the number of Americans who were pedestrians and cyclists on public roads by age. An exposure, like vehicle mile traveled (VMTs), to calculate injury rates would be beneficial, but there is no resource that quantifies VMTs for vulnerable road users to our knowledge. Our choice of decade of life as groups is an attempt to approximate equal populations at risk. We were unable to explore differential risk factors across age groups by race/ethnicity due to concerns about the racial/ethnic information in FARS (Glassbrenner et al. 2022). This study also did not account for socioeconomic status, such as poverty and employment, that can influence the risk of MVC involvement (Glassbrenner et al. 2022). While FARS provides detailed data to the level of county of the crash, no geographic information beyond region is available in CRSS. Future research may expand on this work by augmenting NHTSA data with other data sources such as Census data to investigate risks for vulnerable road users based on social vulnerability index (Centers for Disease Control and Prevention 2023) or socioeconomic indictors of the local areas where they live, such as housing, poverty, unemployment, and transportation, to identify areas in need of resources and tailored interventions.

## Conclusion

Among vulnerable pedestrians and cyclists, children experience differential injury risks due to their limited developmental capacity and their small size to perceive and avoid traffic crashes. Although adults have greater pedestrian and cyclist injury and fatality rates than children, this is not to say that the risk is less for vulnerable child road users. Rather, our results illustrate the unique hazards for younger and older children and in relation to adults, including a greater proportion of pedestrian injuries related to darting out for younger children, and increased cyclist fatality risk for older children due to cyclist midblock-failure to yield. This study identifies risk factors that may be incorporated into practices and programs to improve safety for vulnerable road users, especially child pedestrians and cyclists who require proper supervision. Built environments that promote safety, such as road infrastructure (e.g., sidewalks, one-way streets, greater visibility), traffic calming features (e.g., speed bumps, roundabouts), and reinforcement of traffic laws by all users (pedestrians, cyclists, and drivers) are also necessary to improve safety and prevent injuries among child pedestrians and cyclists.

### Supplementary Information

**Additional file 1. Table S1.** Recategorization of pedestrian and cyclist crash type variables from FARS and CRSS. **Table S2.** Recategorization of FARS and CRSS vehicle type variables.

## Data Availability

The datasets used and analyzed in the current study are available through Fatality Analysis Reporting System (FARS) and Crash Report Sampling System (CRSS), https://www.nhtsa.gov/research-data/fatality-analysis-reporting-system-fars and https://www.nhtsa.gov/crash-data-systems/crash-report-sampling-system, respectively.

## Abbreviations

CI: Confidence intervals
CRSS: Crash Report Sampling System
FARS: Fatality Analysis Reporting System
HCUP: Healthcare Cost and Utilization Project
HHS: Health and Human Services
HRSA: Health Resources and Services Administration
IRB: Institutional review board
MVC: Motor vehicle crash
NHTSA: National Highway Traffic Safety Administration
RR: Risk ratios
VMT: Vehicle mile traveled
