# Supplementary material for: Injury and fatality risks for child pedestrians and cyclists on public roads
Source: Inj Epidemiol. 2024 Apr 11;11:15. doi: 10.1186/s40621-024-00497-2 (PMC11010370; doi:10.1186/s40621-024-00497-2)
Supplement: Supplementary file 1 — Additional file 1. Table S1. Recategorization of pedestrian and cyclist crash type variables from FARS and CRSS. Table S2. Recategorization of FARS and CRSS vehicle type variables. [file 40621_2024_497_MOESM1_ESM.docx]

| **Table S1.** Recategorization of pedestrian and cyclist crash type variables from FARS and CRSS. | |
| --- | --- |
| Pedestrians | |
| Analysis Category | PEDCGP values |
| Crossing | Crossing- No Vehicle Turn; Crossing- Vehicle Turn |
| Dart out | 2016-2019: Dash/Dart out; 2020: Dash - Run, no visual obstruction noted/Dart-out - visual obstruction noted |
| Walk/run along roadway | Walking/Running Along Roadway |
| Unusual circumstances | Unusual Circumstances |
| Other | Backing Vehicle; Working or Playing in Roadway; Bus-related/Bus Stop-related; Unique Midblock; Driveway Access/Driveway Access Related; Waiting to Cross; Pedestrian in Roadway - Circumstances Unknown; Multiple Threat/Trapped; Non-Trafficway; Crossing Expressway; Other/Unknown - Insufficient Details |
|  |  |
| Cyclists | |
| Analysis Category | BIKECGP values |
| Intersection-Motorist failed to yield | Motorist Failed to Yield - Sign-controlled Intersection; Motorist Failed to Yield - Signalized Intersection |
| Intersection-Cyclist failed to yield | Cyclist Failed to Yield - Sign-controlled Intersection; Cyclist Failed to Yield - Signalized Intersection |
| Crossing paths | Crossing Paths - Other Circumstances |
| Midblock-Cyclist failed to yield | Bicyclist Failed to Yield - Midblock |
| Motorist turn | Motorist Left Turn/Merge; Motorist Right Turn/Merge |
| Other | Loss of Control/Turning Error; Parking/Bus-Related; Bicyclist Left Turn/Merge; Bicyclist Right Turn/Merge; Motorist Overtaking Bicyclist; Bicyclist Overtaking Motorist; Wrong-Way/Wrong-Side; Parallel Paths - Other Circumstances; Backing Vehicle; Other/Unusual Circumstances; Non-Trafficway; Other/Unknown - Insufficient Details |

| **Table S2**. Recategorization of FARS and CRSS vehicle type variables. | |
| --- | --- |
| Category | Vehicle type |
| Cars | Convertible, 2-door sedan, 3-door/2-door hatchback, 4-door sedan, 5-door/4-door hatchback, station wagon, hatchback, sedan/hardtop, other automobile type, 3-door coupe, auto based pickup, auto based panel, three-wheel automobile derivative |
| Vans, utility vehicles, light trucks | Compact utility, large utility, utility station wagon, utility vehicle unknown, minivan, large van, step/walk-in van, other/unknown van, compact pickup, standard pickup, pickup with slide-in camper, convertible pickup, light pickup, unknown light conventional truck, cab chassis based, truck-based panel, other light conventional truck, unknown light truck/vehicle type |
| Buses, trucks | School bus, cross country/intercity bus, transit bus, van-based bus, other/unknown bs type, step van, single-unit straight truck or cab-chassis (GVWR: 10,000-19,500; 19,501-26,000; > 26,000), truck-tractor, medium/heavy pickup, unknown truck type, motor homes |
| Motorcycles, other | Motorcycle/2-wheel motorcycle, moped/motorized bike, 3-wheel motorcycle, off-road motorcycle, motor scooter, unenclosed/enclosed/unknown 3-wheel motorcycle, other motorcycle type, ATV, snowmobile, construction equipment, golf cart, recreations off-highway vehicle, go-cart, fork-lift, street sweeper, not reported, unknown body type |
| Note: Categories guided by National Center for Statistics and Analysis (2022a) report. | |
